# Orbital metastasis of invasive lobular carcinoma of the breast

**DOI:** 10.1093/jscr/rjab619

**Published:** 2022-01-21

**Authors:** Shinichi Tsutsui, Koto Kawata, Tsutomu Ubagai, Satoshi Okimoto, Megumu Fujihara, Takashi Maeda, Takashi Sonoda

## Abstract

We herein report a case of orbital metastasis from the breast cancer in a 58-year-old woman presenting with visual disturbance and bilateral periorbital swelling. She had undergone radical mastectomy for right breast cancer 9 years previously and been receiving hormone therapy for bone metastasis of breast cancer for the past 4 years. Computed tomography and magnetic resonance imaging revealed an ill-defined mass in the bilateral orbits, whereas an excisional biopsy confirmed metastasis of invasive lobular carcinoma (ILC) of the breast. The appearance of eye symptoms in patients who have a history of breast cancer, especially ILC should be investigated, with a consideration of orbital metastasis.

## INTRODUCTION

Metastasis to the orbit is a rare manifestation of metastatic cancer [1, 2]. A review of 1264 patients with orbital tumors [3] indicated metastatic tumors to account for only 9% (91/1264 cases), whereas the breast (44/91 cases), followed by the prostate, lung, skin (melanoma), kidney and gastrointestinal tract, were the most common primary sites of cancers that metastasized to the orbit [1, 3]. The clinical symptoms of orbital metastasis, such as disturbance in ocular mobility, proptosis, diplopia, pain and periorbital swelling, are similar to those of other orbital tumors, such as idiopathic pseudotumor and IgG4-related orbital disease [4].

## CASE REPORT

**Figure 1:**
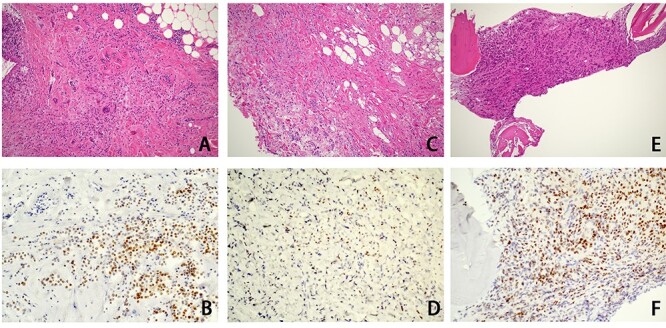
Histological examination findings for ILC of the breast. An hematoxylin–eosin examination of primary breast cancer that had been resected 9 years earlier showed ILC of the breast (**A**), and ILC was also shown in the biopsy specimens of the orbit (**C**) and bone marrow (**E**). Immunohistochemical studies showed positive ER expression in the primary breast cancer (**B**) and biopsy specimen of bone marrow (**F**), whereas the ER expression of the biopsy specimen of the orbit (**D**) was negative.

A 58-year-old woman had undergone radical mastectomy for right breast cancer 9 years earlier. A histological examination of the primary breast cancer revealed invasive lobular carcinoma (ILC) (Fig. 1A), whereas an immunohistochemical study indicated estrogen receptor (ER) positivity (Fig. 1B) and progesterone receptor (PgR), Her2 and E-cadherin negativity. She underwent postoperative adjuvant chemotherapy, followed by hormone therapy. Since bone metastasis of the rib occurred 4 years ago, high-dose toremifene citrate was indicated. Hormone therapy was then switched to fulvestrant 3 years ago and abemaciclib was also added to fulvestrant 6 months ago.

**Figure 2:**
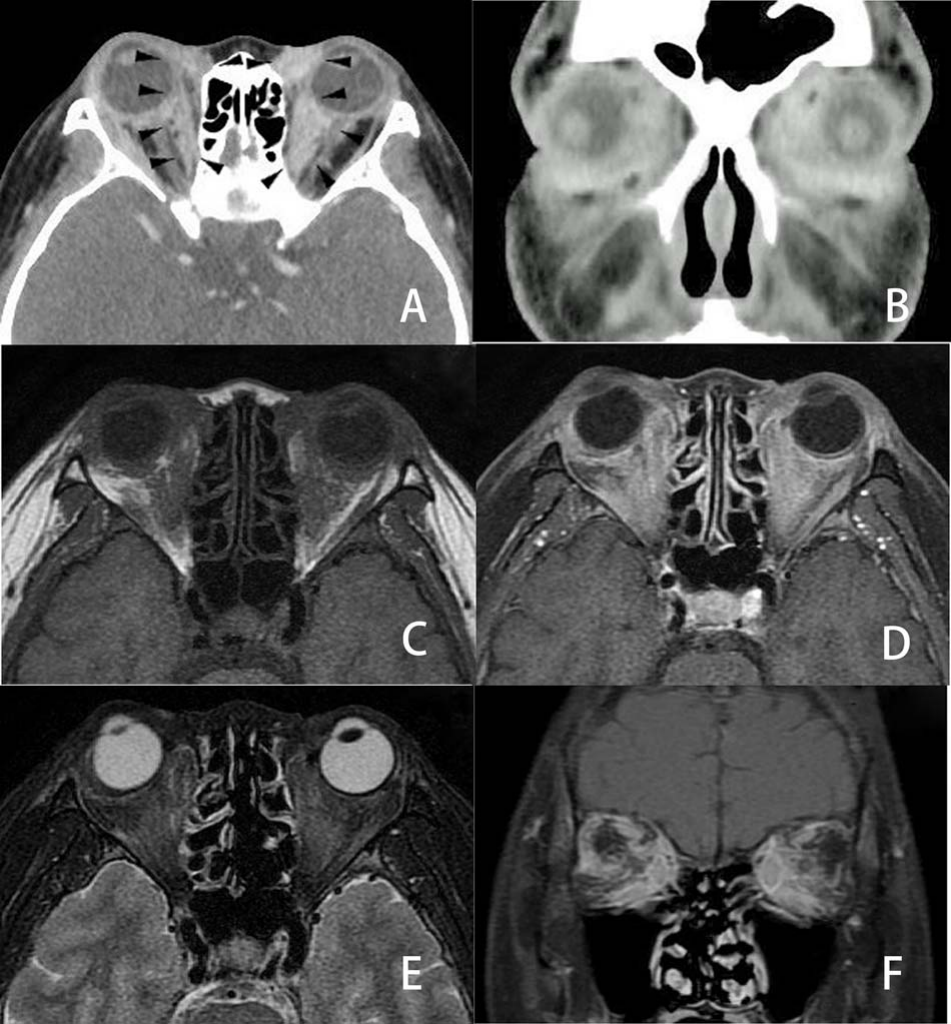
Three-dimensional imaging findings of orbital metastasis from breast cancer. Axial (**A**) and coronal (**B**) CT with contrast showed abnormal soft tissue enhancement (arrow) along the eyeball in the medial to retrobulbar portions of the bilateral orbits. The enhancement of the left orbit was greater than that of the right orbit. Axial MRI (**C**, **D**, **E**) also revealed an ill-defined tumor involving the soft tissues at the same portion where the soft tissue enhancement was seen on CT in the bilateral orbits. These orbital tumors were hypointense to fat tissue on axial T1-weighted imaging (C) and slightly enhanced on axial T1-weighted imaging with contrast (D) but slightly hyperintense to fat tissue on axial fat-suppressed T2-weighted imaging (E). Coronal fat-suppressed T1-weighted imaging with contrast (**F**) showed heterogenous irregular enhancement in the retrobulbar portion of the bilateral orbits. CT and MRI revealed no destruction of the eyeball, optic nerve or orbital bony wall.

She complained of visual disturbance and bilateral periorbital swelling. An ophthalmological examination revealed limited upturn and abduction movement in the bilateral eyes. Computed tomography (CT) revealed abnormal soft tissue enhancement along the eyeball in the medial to retrobulbar portions of the bilateral orbits (Fig. 2A and B). Magnetic resonance imaging (MRI) (Fig. 2C–F) further indicated an ill-defined mass at the same portion where the soft tissue enhancement had been shown by CT in the bilateral orbits. These orbital tumors were hypointense to fat tissue on T1-weighted imaging but slightly hyperintense to fat tissue on T2-weighted imaging. T1-weighted imaging with contrast showed heterogenous irregular enhancement in the retrobulbar portion of the bilateral orbits. CT and MRI also indicated no destruction of the orbital wall due to bone metastasis. Positron emission tomography indicated a slight uptake in the bilateral orbits, with no significant uptake except for in the bone and orbital space. Since CT and MRI could not differentiate metastatic tumors from inflammatory tumors, such as IgG4-related disease, a *trans*-palpebral biopsy confirmed the metastasis of ILC (Fig. 1C), which was identical to the primary breast cancer. Thrombocytopenia simultaneously occurred and a biopsy of bone marrow also confirmed metastasis of breast cancer to the bone marrow (Fig. 1E). Immunohistochemical studies demonstrated negative findings for ER (Fig. 1D), PgR and Her2 for orbital metastasis but ER positivity (Fig. 1F) and PgR and Her2 negativity for the bone marrow metastasis.

The patient ultimately died from pancytopenia due to bone marrow dissemination 3 months later.

## DISCUSSION

A variety of tumors and pseudotumors may involve the orbit [4]. Orbital inflammatory pseudotumor is an idiopathic tumor-like inflammation, such as autoimmune thyroid disease, sarcoidosis, lymphoproliferative disease and IgG4-related disease [4]. Three-dimensional CT and MRI are useful for detecting the presence of these tumors [5]. Characteristic imaging features that might help distinguish among these orbital tumors and metastatic tumor was reported to include hypointensity to fat on T1-weighted imaging and hyperintensity to fat on T2-weighted imaging [6]. However, determining the etiology of an orbital tumor by radiological imaging alone is difficult, as radiological imaging findings of orbital tumors usually overlap each other [5–8]. Therefore, an excisional biopsy is necessary to confirm the histological diagnosis of orbital tumors [5–8].

Invasive ductal carcinoma (IDC) is the major histological type in breast cancer, with ILC accounting for about 10% of primary breast cancers [9]. Although there is no significant difference in prognostic features between ILC and IDC, ILC is known to have different metastatic features from IDC [9]. ILC is associated with an increased incidence of bone metastasis and is known to preferentially involve uncommon sites, such as the peritoneum, uterus, ovary, gastrointestinal tract and skin [9]. Raap *et al*. [7] reported 14 cases of orbital metastasis and reviewed 72 cases from 68 independent case reports. The primary lesions of orbital metastasis were breast in 8 of 14 cases, and the histological diagnosis was ILC in 7 of those 8 cases. Furthermore, breast cancer was the most common primary site (21/72, 29%), and the histological subtype was ILC in 11 cases and IDC in 2 cases. Another review of 57 cases with orbital metastasis from breast cancer indicated that the histological types were ILC in 12 cases and IDC in 5 cases and poorly differentiated or undifferentiated breast cancer in 7 cases; the histological details were lacking in the remaining 34 cases [8]. Given the relative rarity of ILC in primary breast cancer, there is a remarkably high frequency of ILC in orbital metastasis. Although a loss of E-cadherin expression in ILC has been thought to be associated with the differences in the metastatic pattern of ILC from IDC, the detailed mechanism remains unclear [7]. On the other hand, estrogen-rich conditions are suggested to be one reason for the higher rate of orbital metastasis in ILC than in IDC [8, 10].

We previously reported the differences in the biological parameters between primary and metastatic lesions [11] and between primary and recurrent lesions [12]. Thirteen metastatic lesions (22%) of the 60 primary lesions with ER positivity changed ER negativity [11]. The loss of ER expression in recurrent lesions is thought to be due to systemic therapy [13]. In the present study, however, the orbital metastasis was negative for ER, whereas the bone marrow metastasis was positive for ER, the same as for the primary breast cancer. Our earlier findings suggested the selection mechanisms from the primary lesion to the metastatic lesion of esophageal cancer [14]. The intratumoral heterogeneity with clonal selection is thought to be one reason for the difference in the protein expression in primary, metastatic and recurrent lesions of cancer [15].

## CONCLUSION

The appearance of eye symptoms, such as visual disturbance, periorbital swelling and proptosis, in patients with a history of breast cancer, especially ILC, should be investigated with suspicion of orbital metastasis. Three-dimensional CT and MRI should be performed to detect the presence of an orbital tumor, but a biopsy is necessary to confirm the histological diagnosis of orbital metastasis from breast cancer.
